# Mesoangioblast delivery of miniagrin ameliorates murine model of merosin-deficient congenital muscular dystrophy type 1A

**DOI:** 10.1186/s13395-015-0055-5

**Published:** 2015-09-03

**Authors:** Teuta Domi, Emanuela Porrello, Daniele Velardo, Alessia Capotondo, Alessandra Biffi, Rossana Tonlorenzi, Stefano Amadio, Alessandro Ambrosi, Yuko Miyagoe-Suzuki, Shin’ichi Takeda, Markus A. Ruegg, Stefano Carlo Previtali

**Affiliations:** Institute of Experimental Neurology (INSPE) and Division of Neuroscience, IRCCS San Raffaele Scientific Institute, Via Olgettina 60, 20132 Milano, Italy; Tiget and Division of Regenerative Medicine, IRCCS San Raffaele Scientific Institute, Milano, Italy; University San Raffaele Vita e Salute, Milano, Italy; Department of Molecular Therapy, National Institute of Neuroscience, National Center of Neurology and Psychiatry, Ogawa-higashi, Kodaira, Tokyo Japan; Biozentrum, University of Basel, Basel, Switzerland

**Keywords:** Mesoangioblast, Congenital muscular dystrophy, Laminin, Miniagrin, Therapy

## Abstract

**Background:**

Merosin-deficient congenital muscular dystrophy type-1A (MDC1A) is characterized by progressive muscular dystrophy and dysmyelinating neuropathy caused by mutations of the α2 chain of laminin-211, the predominant laminin isoform of muscles and nerves. MDC1A has no available treatment so far, although preclinical studies showed amelioration of the disease by the overexpression of miniagrin (MAG). MAG reconnects orphan laminin-211 receptors to other laminin isoforms available in the extracellular matrix of MDC1A mice.

**Methods:**

Mesoangioblasts (MABs) are vessel-associated progenitors that can form the skeletal muscle and have been shown to restore defective protein levels and motor skills in animal models of muscular dystrophies. As gene therapy in humans still presents challenging technical issues and limitations, we engineered MABs to overexpress MAG to treat MDC1A mouse models, thus combining cell to gene therapy.

**Results:**

MABs synthesize and secrete only negligible amount of laminin-211 either in vitro or in vivo. MABs engineered to deliver MAG and injected in muscles of MDC1A mice showed amelioration of muscle histology, increased expression of laminin receptors in muscle, and attenuated deterioration of motor performances. MABs did not enter the peripheral nerves, thus did not affect the associated peripheral neuropathy.

**Conclusions:**

Our study demonstrates the potential efficacy of combining cell with gene therapy to treat MDC1A.

**Electronic supplementary material:**

The online version of this article (doi:10.1186/s13395-015-0055-5) contains supplementary material, which is available to authorized users.

## Background

Merosin-deficient congenital muscular dystrophy type 1A (MDC1A; OMIM #607855) is a severe and progressive muscle-wasting neuromuscular disease that frequently leads to death in early childhood. The disease is inherited as autosomal recessive and is characterized by muscular dystrophy, dysmyelinating neuropathy, and minor brain abnormalities [1, 2]. MDC1A is caused by mutations in the *LAMA2* gene, which encodes the α2 chain of laminin-211 (or merosin), the major component of the basement membrane of muscles and peripheral nerves [3]. Mutations result in loss of interaction with laminin-211 receptors expressed by striated muscle and Schwann cells, primarily integrin α7β1, α6β1, and dystroglycan [4–6], thus leading to progressive tissue degeneration and ultimately to muscular dystrophy and neuropathy [7–13]. Several mouse models for MDC1A are available: the spontaneous mutant dy^2J^/dy^2J^ (abbreviated as dy^2J^, this point on) resulting in a truncated protein, which displays a mild phenotype [14–16]; the complete null mutant dy^3K^/dy^3K^ (abbreviated as dy^3K^, this point on), which has a severe phenotype [17], and the dy^W^/dy^W^ mutant, a mouse that still synthesizes a very small amount of truncated laminin α2 chain [18].

There is currently no therapy to treat MDC1A. However, in the last years, promising therapeutic attempts have been carried out using mouse models. Recent evidence showed that overexpression of a miniaturized form of agrin, miniagrin (MAG), which binds to dystroglycan but not integrin α7β1, ameliorates the disease in MDC1A mouse models [19–21]. In fact, MAG acts as a linker between dystroglycan and other laminin isoforms (laminin-411 and -511), which are overexpressed in MDC1A but cannot bind efficiently to dystroglycan [3, 19, 22, 23]. Along with transgenic overexpression of MAG, engineered adeno-associated viral (AAV) vector to systemically deliver MAG showed similar efficacy to ameliorate muscular dystrophy in MDC1A mouse model [24]. However, although these data point the way to a promising new therapeutic approach for MDC1A, direct gene therapy in humans still presents challenging technical issues and limitations in terms of safety and efficacy [25, 26].

Cell therapy has been considered a suitable and more feasible approach for treatment of human neuromuscular disorders, either when it has been used for tissue replacement [27, 28] or as a carrier vehicle to deliver protein of interest [29, 30]. Mesoangioblasts (MABs) are vessel-associated progenitors [31], which can be isolated from mesodermal tissues and expanded in vitro. MABs repopulate the skeletal muscles when injected into the blood stream or directly into the muscles. MABs have been shown to restore to a significant extent muscle structure and function in animal models of muscular dystrophy [32–36], and based on this preclinical evidence on safety and efficacy, a clinical trial with allogenic MABs transplanted in patients with Duchenne muscular dystrophy has been performed at the San Raffaele Scientific Institute in Milan (EudraCT no. 2011-000176-33).

Here, we show that by combining MAB cell therapy with MAG delivery, we ameliorated the phenotype of MDC1A mouse models. MABs were engineered to produce mouse MAG (mMAG) and were delivered into adult dy^2J^ mice. Treated mice showed diffuse expression of mMAG at the sarcolemma surface and increased expression of laminin-211 receptors. Significant amelioration of muscle histology and reduced deterioration of motor performances were observed, whereas no effects on peripheral neuropathy were noted. This is one of the first cell therapy approaches to MDC1A, and our findings suggest a novel feasible strategy to treat MDC1A with realistically fast translation into clinical practice.

## Methods

### Mice

All the experiments received ethical approval and were performed in agreement with the Ospedale San Raffaele Institutional Animal Care and Use Committee (IACUC authorization #487 and #664). The dy^2J^/dy^2J^ (C57BL/6J background) and NOD SCID (NOD.CB17-Prkdc^SCID^/J or SCID; NOD/ShiLtSz background) mice were purchased from Jackson Laboratories (Bar Harbor, USA). The dy^3K^/dy^3K^ (C57BL/6J background) mice were previously described [17]. Both dy^2J^/dy^2J^ and dy^3K^/dy^3K^ mice were maintained in the C57BL/6J background; double dy^2J^/dy^2J^//NOD SCID mice (abbreviated as SCIDdy^2J^, this point on) were in mixed background at F2/F3 generation. For routine genotyping, we isolated genomic DNA from tail biopsies, using DirectPCR solution (Viagen), according to the manufacturer’s directions. Primer sequences are available upon request.

### Cell cultures

Clone D16 and C57 of mouse MABs were previously described [34, 37]. MABs and MABs carrying miniagrin (MABs + mMAG) were maintained in culture in Dulbecco’s modified Eagle’s medium (DMEM, high glucose; Invitrogen) supplemented with 20 % of heat-inactivated fetal bovine serum (FBS; EuroClone), 2 mM glutamine, 1 mM sodium pyruvate, 100 IU ml^−1^ penicillin, and 100 μg ml^−1^ streptomycin (Invitrogen) in 5 % CO_2_ humidified atmosphere. Myogenic differentiation of MABs was induced by plating 75 × 10^3^ cells onto 0.1 % poly-l-lysine hydrobromide, collagen (1 mg/ml)-coated dishes (Sigma). Differentiation medium consisted of DMEM supplemented with 2 % horse serum (Sigma), 2 mM glutamine, 1 mM sodium pyruvate, 100 U ml^−1^ penicillin, and 100 μg ml^−1^ streptomycin (Invitrogen). Cultures were incubated at 37 °C, 5 % CO_2_ for different periods (5–10 days) and then processed for immunofluorescence analysis. Human MABs were obtained from healthy donors as described in [37].

### Mesoangioblast treatment with 5-azacytidine and trichostatin A

Inhibition of DNA methyl-transferase and/or histone deacetylase was performed in proliferating or differentiated murine MABs. For proliferation conditions, MABs were plated in proliferation medium (DMEM supplemented with 15 % fetal bovine serum (Invitrogen) at 1.2 × 10^3^ cells/cm^2^ density on 6-well multiwells. 5-Aza-2′deoxycytidine (AZA) was added to 1 uM final concentration every 24 h for 3 days. Trichostatin A (TSA) was added to 1 uM final concentration on day 3, 12–15 h before collecting cells for analysis. For differentiation conditions, MABs were plated in proliferation medium at 1.2 × 10^3^ cells/cm^2^ density on collagen (1 mg/ml) coated 6-wells multiwells (dilution 1:100). AZA was added to 1 uM final concentration every 24 h for 5–7 days, shifting to differentiation medium (DMEM supplemented with 2 % horse serum; Invitrogen) on day 3. TSA was added to 1 uM final concentration on days 5–7 (differentiation of cells should be achieved), 12–15 h before collecting cells for analysis.

### Quantitative RT-PCR

Total RNA was isolated from murine-differentiated MABs using TriPure Isolation Reagent (Roche) according to the manufacturer’s instructions. In brief, cells were homogenized in the presence of TriPure Isolation Reagent, and total RNA was extracted with chloroform and precipitated with isopropanol. Two micrograms of total RNA was reverse transcribed using a High-Capacity cDNA Reverse Transcription kit (Applied Biosystems). Quantitative RT-PCR analyses were performed on a 7900HT Real-Time PCR System using the 2× TaqMan PCR Mastermix (Applied Biosystems) according to manufacturer’s recommendations. The primers used were TaqMan Gene Expression Assays ID: Mm01203489_g1 for *MyoD1*, Mm00446195_g1 for *MyoG*, and Mm99999915_g1 for *Gapdh*. Levels of gene expression were determined with the comparative cycle threshold (ΔΔCt) method. The mRNA level of *Myod1 and MyoG* gene was normalized to the level of *Gapdh* mRNA.

Each time point is the mean of three experiments.

### Mesoangioblast transplantation

Thirty dy^2J^ mice were immunosuppressed using daily injection of tacrolimus (FK506 at 2.5 mg/kg; Astellas), started 3 days before the first cell injection (42 days of age). We then injected saline, MABs, or MABs + mMAG (ten mice per group, see scheme in Fig. 5a) into the tibialis anterior, gastrocnemius, and quadriceps muscles of dy^2J^ mice, starting at 45 days of age. Only before this first cell (or saline) injection, all the dy^2J^ mice were treated (intramuscular injection) with 20 μg of cardiotoxin (CTX from *Naja mossambica mossambica*; Sigma; 0.5 μg/μl, 40 μl vol. per muscle), injected 24 h before MAB/saline. Prior to any procedure, mice were anesthetized by intraperitoneal injection of avertin (2,2,2-tribromoethanol, Sigma-Aldrich) in 0.9 % saline solution. Intramuscular delivery was performed by the injection of approximately 1 × 10^6^ MABs or MABs + mMAG using a 30-gauge needle. Control mice (dy^2J^ not treated) were injected only with saline solution. All dy^2J^ mice received saline, MABs, or MABs + mMAG (same dosage) at 55, 65, and 75 days of age (Fig. 5a) and were analyzed for treadmill and histology at 85 days of age. Dy^3K^ mice were treated as described above at 18 days of age and analyzed 7 days after MAB injection.

### Histology and immunohistochemistry

Muscle samples were rapidly frozen in liquid nitrogen cooled isopentane (Sigma). Muscle histology and hematoxylin and eosin (H&E; Bio-Optica) staining was performed according to standard laboratory protocol. Immunofluorescence was performed as previously described [38] and examined with confocal (Leica SP5, Leica Microsystems) and fluorescence (Olympus BX51) microscopes. For cell cultures, cells were grown on onto 0.1 % poly-l-lysine hydrobromide-coated glass coverslips, washed with PBS, and fixed with 4 % paraformaldehyde at room temperature for 10 min. After the incubation with the primary antibody, cells or tissue sections were washed in PBS, incubated with appropriate FITC- or TRITC-conjugated anti-rabbit, anti-rat or anti-mouse antibody for 30 min at RT, washed in PBS, and then mounted on glass slides with Vectashield Mounting Medium (Vector Laboratories).

Sirius Red staining for collagen deposition was performed as follows: muscle cryosections were treated overnight with Bouin solution (picric acid, formaldehyde, and 5 % acetic acid), washed and then incubated for 1 h in 1 % direct red solution and picric acid; washed in 2 % acetic acid and then in 1:1 solution ethanol/picric acid, dehydrated, cleared in xylene, and coverslipped with mounting medium.

For quantification, digitalized images were collected using light Olympus (BX51) microscope and digital camera (DFC300F Leica Microsystems). Muscle fiber size distribution and number of centrally located nuclei was determined on H&E-stained tibialis anterior muscle cross-sections by means of the QWin software (Leica Microsystems) as described [38]. At least four random images (×10 objective), representing the dystrophic areas of histologic mid-belly muscle sections from five different mice per genotype were analyzed, corresponding to a minimum of 5000 muscle fibers per mouse. Quantification of muscle fibrosis was performed by using ImageJ software (NIH) on Sirius Red-stained cryosections, four random images (×10 objective) representing the dystrophic areas of histologic mid-belly muscle sections from five different mice per genotype. Sirius Red-stained area was calculated as a percentage of total muscle area analyzed.

Peripheral nerve semithin sections were performed as described [39], stained with toluidine blue, and examined by light (Olympus BX51) and electron (CEM 902; Carl Zeiss) microscopy.

### Western blot

Cells were homogenized in protein extraction buffer (50 mM Tris-HCl, pH 7.4, 150 mM NaCl, 5 mM EDTA, 1 % triton supplemented with proteases inhibitors). Protein concentrations were determined by Bradford protein assay (Bio-Rad). Twenty micrograms of proteins were loaded and separated on a 10 % SDS-PAGE and immunoblotted for the detection of mMAB protein, except for the detection of laminin α2 in the experiments with trichostatin and AZA, in which we loaded 150 μg of proteins per lane to be able to detect a minimal amount of laminin α2. Muscles were powdered in liquid nitrogen using a mortar and a pestle. Protein extraction was completed in ice-cold lysis buffer containing: 75 mM Tris-HCl pH 6.8, 1 % SDS supplemented with proteases inhibitors (Complete, Roche), and when needed, phosphatase inhibitors (PhosSTOP, Roche). Then, the samples were sonicated and centrifuged for 5 min at 13,000 rpm. Supernatants were collected and protein concentrations were quantified using the BCA protein assay (Pierce). Twenty micrograms of proteins of total homogenates were loaded and separated on 8–12 % SDS poliacrylamide gels at 80 V for 3 h. Proteins were then transferred to nitrocellulose membranes (Bio-Rad) at 35 V O/N at 4 °C, and membranes were stained with Ponceau (Sigma) to ensure equal protein loading. Membranes were saturated with 5 % milk in 0.1 % Tween-20 (Sigma) PBS for 1 h at RT and hybridized for 2 h at RT with the following primary antibody. Detection was carried out by incubation with horseradish peroxidase-conjugated anti-rabbit or anti-mouse IgG (Santa Cruz Biotechnology) for 1 h at RT. The proteins were visualized by ECL immunoblotting detection system (Amersham). Densitometric analyses were performed by using the Scion Image software. Averages of densitometric measurements of independent experiments, normalized by the endogenous β-actin or β-tubulin values, were compared by Student's *t* test. The results shown in the figures are representative of three separate experiments. Data are expressed as means ± SEM.

### Antibodies

Antibodies used for immunohistochemistry and/or Western blot analysis included (p = polyclonal, m = monoclonal, Ch = chicken, Ms = mouse, Rb = rabbit, Gt = goat, Rt = rat): α-actin (mMs Sigma); calnexin (pRb; Sigma C4731); β-dystroglycan (mMs, 8D5, Novocastra); α-dystroglycan (mMs, nIIH6C4, Millipore) and α-dystroglycan (mMs, VIA4-1, Millipore); integrin α5 (pRb, AB1949, Millipore); integrin a6 (mRt, GoH3 from A. Sonnenberg); integrin α7 (pRb, from Dr. U. Mayer); β1 integrin (pRb; Millipore); laminin α1 (mRt, AL-1, Millipore); laminin α2 (mMs; Alexis 4H8-2) and laminin α2 (mMs; 1:200, gift from H. Hori); laminin α4 (pRb, H-194, Santa Cruz); laminin α5 (pRb, 405, from Dr. Sorokin); laminin γ1 (mRt, MAB1914, Millipore); laminin HSA (pRb, L9393, Sigma); myc tag (mMs; Cell Signaling); myosin heavy chain (mMs, Hybridoma Bank); neurofilament M (pCh; 1:1000, Covance PKC-593P); and β-tubulin (mMs; TUB2.1, Sigma).

### TUNEL assay

Terminal deoxynucleotidyl transferase dUTP nick end labeling (TUNEL) assay was performed as described [39], using the fluorometric TUNEL System G3250 Kit (Promega). Briefly, cryosections (10 μm thick) of quadriceps muscles were fixed in 4 % paraformaldehyde, permeabilized in 0.1 % triton, treated with DNase solution, and finally DNA strand breaks labeled with fluorescein-12-dUTP by deoxynucleotidyl transferase (TdT)-reaction mix for 60 min before the stop reaction. Nuclei were identified by DAPI and muscle fibers by anti-total-laminin antibody. For quantification, TUNEL/DAPI double positive nuclei associated with single muscle fiber were counted and expressed as the percentage of total number of positive fibers; we counted at least 1600 fibers per mouse, three mice per genotype.

### Cell transfection with lentiviral vectors

Lentiviral production and transfection was performed as previously described [29]. Briefly, 3.2 kb of mMAG construct (including a myc tag) was sequenced and subcloned downstream of the uni-directional hPGK promoter of the pCCLSIN.PPT.hPGK.wPRE lentiviral vector (LV). Concentrated mMAG LV stocks pseudotyped with the VSV.G envelope were produced by transient co-transfection of four plasmids in 293T cells and titreted on HeLa cells. MABs were transduced at multiplicity of infection (MOI) of 100 with mMAG LV or control vector. Ten days after transduction, MABs were collected and proteins secreted in cell culture medium were precipitated to evaluate the expression of mMAG. The presence of mMAG was tested by Western blot using anti-myc tag antibody.

### Treadmill exercise

Functional muscle activity was measured by using the exhaustion treadmill to assess resistance to fatigue. For the pretreatment exercise test, 30 45-day-old dy^2J^ mice were tested for functional performance with the treadmill test (Columbus Instruments, Columbus, OH, USA). MAB-treated and not-treated mice were retested for functional recovery at 85 days (Fig. 5a). For the exercise test, mice were put into treadmill at 3 m/min, then the speed was increased 1 m/min every 2 min until exhaustion. Final value is the mean (±SEM) of three different exercises per mouse. Mice were adapted to the procedure (10 min every other day; 3 m/min) for 1 week before beginning the exercise training protocol. The examiner was blind as respect to the scheme of treatment. Deterioration was expressed in percentage and measured as: B-A/A × 100; *B* = average time on treadmill post-treatment, *A* = average time on treadmill before treatment.

### Neurophysiology

Neurophysiology was performed as described [38]. The dy^2J^ mice were analyzed before (45 days of age) and after (85 days of age) MAB treatment (see scheme in Fig. 5a). Mice were anesthetized with tribromoethanole (Avertine; Sigma) and placed under a heating lamp to avoid hypothermia. Sciatic nerve conduction velocities (NCV) were obtained by stimulating the nerve with steel monopolar needle electrodes. A pair of stimulating electrodes was inserted subcutaneously near the nerve at the ankle, and then they were moved to the sciatic notch to obtain two distinct sites of stimulation, proximal and distal along the nerve, in order to obtain the measurement of NCV between them. The muscular response to the electrical nerve stimulation (compound motor action potentials (cMAP)) was recorded with a pair of needle electrodes; the active electrode was inserted in muscles in the middle of the paw, while the reference was placed in the skin between the first and second digit. The cMAP peak-to-peak amplitude was considered for analysis. The examiner was blind as respect to the scheme of treatment.

### Statistical analysis

Values were expressed as means ± SEM. Significance of the differences was assessed between means by two-tailed unpaired Student’s *t* test or ANOVA with Tukey’s *post hoc* test (GraphPad Prism software version 5). We investigated the differences between the distributions by means of the Mann-Whitney test and verified for stochastic dominance. To take into account of test multiplicity, *p* values were adjusted for false discovery rate [40]. A probability of less than 5 % (*p* < 0.05) was considered to be statistically significant.

## Results

### Mesoangioblasts do not express laminin-211 but laminin-411 and laminin-511

**Fig. 1:**
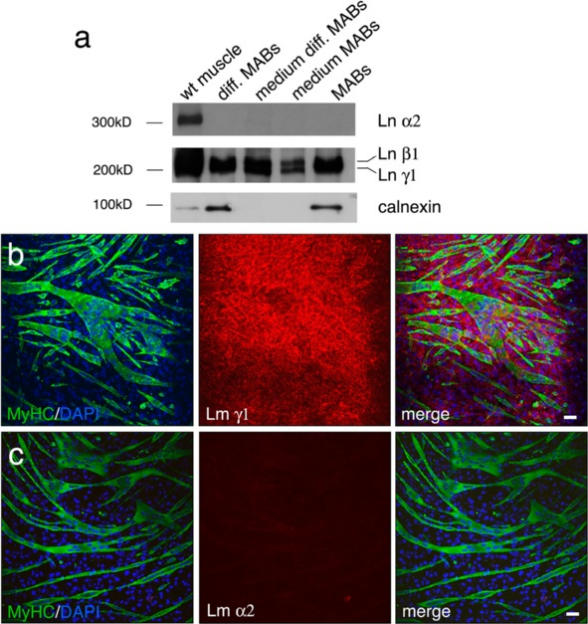
MABs do not synthesize laminin α2 neither in vitro nor in vivo. **a** Western blot analysis of mouse wild type (C57Bl6) muscle, differentiated and undifferentiated MABs, and medium from differentiated and undifferentiated MABs. Anti-laminin α2 chain antibody detects a specific band only in wild type muscle homogenate, whereas a polyclonal antibody recognizing laminin chain β1 and γ1 detects specific bands in all the homogenates, including medium from differentiated and undifferentiated MABs. Calnexin is shown as loading control (accordingly, it is absent when only medium is loaded). Each lane is loaded with 40 μg of protein. **b** Cultures of MABs differentiated in myotubes, as depicted by positive staining with anti myosin heavy chain (MyHC) antibody, show positive staining for laminin chains γ1. DAPI staining identifies nuclei. Scale bar = 30 μm. **c** Cultures of MABs differentiated in myotubes (MyHC positive) do not show staining with anti-laminin α2 chain antibody. DAPI staining identifies nuclei. Scale bar = 30 μm

**Fig. 2:**
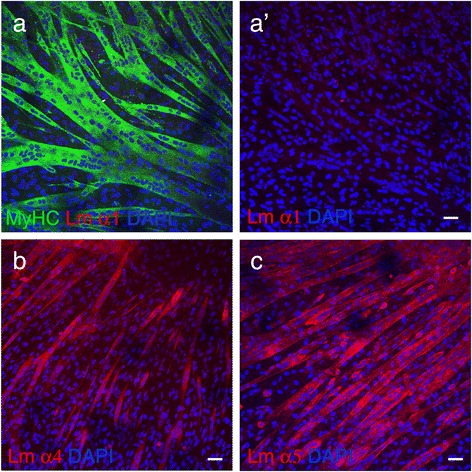
MABs can synthesize laminin chains α4 and α5 but not α1. **a** Cultures of MABs differentiated in myotubes (MyHC positive) do not show staining with anti-laminin α1 chain antibody. DAPI staining identifies nuclei. **a′** is the same image as in (**a**), without MyHC staining. Scale bar = 30 μm. **b** Cultures of MABs differentiated in myotubes show positive staining for laminin α4. DAPI staining identifies nuclei. Scale bar = 30 μm. **c** Cultures of MABs differentiated in myotubes show positive staining for laminin α5. DAPI staining identifies nuclei. Scale bar = 30 μm

Previous studies showed that MABs either directly transplanted in the skeletal muscle or injected into the blood stream can engraft dystrophic muscles and restore the expression of muscular proteins of different types of dystrophic mice [32–36, 41]. In order to evaluate if MABs may be useful to ameliorate MDC1A, we first investigated if MABs can synthesize and express laminin-211. MABs (cell line C57 and D16) were grown undifferentiated or were differentiated to myotubes, and the expression of laminin α2, β1, and γ1 was investigated by Western blot analysis and immunohistochemistry. We did not detect any laminin α2 chain either in cell homogenate or medium of both undifferentiated and differentiated MABs (Fig. 1a). Conversely, we detected both laminin chains β1 and γ1 (Fig. 1a). Similarly, immunohistochemistry showed that MABs, either undifferentiated or differentiated to myotubes (MyHC positive), stained positive with polyclonal antibody recognizing laminin chains β1 and γ1 but not with specific monoclonal antibody recognizing laminin α2 (Fig. 1b, c). We then evaluated if MABs can synthesize other laminin isoforms expressed in the muscle and nerve and observed in MDC1A mutants [3, 4, 19, 23]: we detected by immunohistochemistry laminin chains α4 and α5 but not α1 (Fig. 2a–c). Thus, MABs can assemble laminin-411 and -511 but not -211 and -111.

**Fig. 3:**
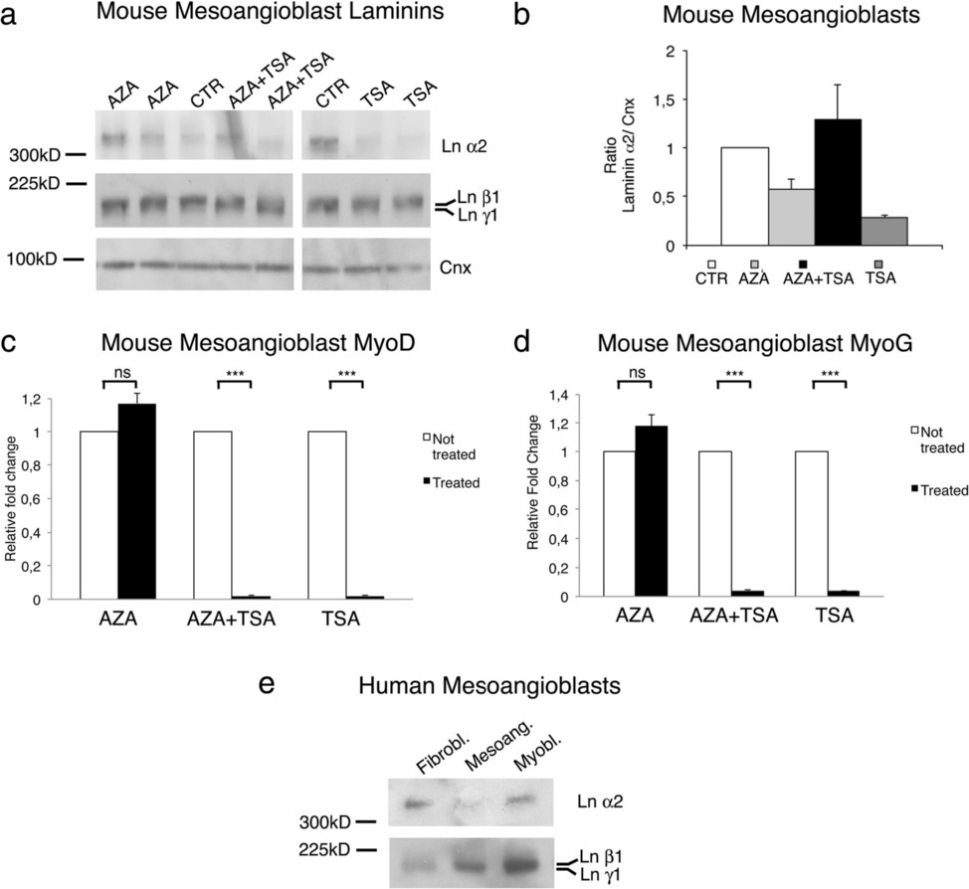
Human MABs and mouse MABs treated with deacetylating and demethylating substrates express very little amount of laminin α2 chain. **a** Western blot analysis of homogenates from differentiated mouse MABs (150 μg of protein loaded per lane) untreated (CTR) or treated with 5-aza-2′deoxycytidine (*AZA*), AZA and trichostatin A (AZA + TSA), or trichostatin A (*TSA*) for 3 days. Deacetylation (AZA) and/or demetylation (TSA) do not modify the expression of laminin chain a2, β1, and γ1. Calnexin (*Cnx*) is shown as reference for protein loading. **b** Quantification of densitometric results of the above the Western blot analysis as ratio between laminin chain α2 and calnexin, representing the mean of three determinations (±SEM). **c**–**d** Quantitative PCR analysis for MyoD (**c**) and MyoG (**d**) of differentiated MABs untreated or treated with AZA, AZA + TSA, or TSA; as expected MyoG was significantly increased by AZA and decreased by TSA treatment. **e** Western blot analysis of homogenates from human fibroblasts, human MABs, and human myoblasts. One hundred micrograms of protein were loaded per lane. Anti-laminin α2 chain antibody detects positive band in fibroblasts and myoblasts, whereas very little (if any) positive staining is present in human MABs. All three homogenates stained positive for laminin chain β1 and γ1

To evaluate whether reduced capacity of MABs to synthesize laminin α2 was due to epigenetic events, we tested if histone deacetylase and/or methyl-transferase inhibitors could modify the expression of laminin α2 in murine MABs. We treated undifferentiated and differentiated C57 murine MABs with 5-aza-2′deoxycytidine (AZA; to inhibit histone deacetylase), trichostatin (TSA; to inhibit methyl-transferase), or both AZA + TSA. To detect minimal amount of laminin α2, we overloaded samples (150 μg per lane) and overexposed the membrane stained with anti α2 antibody. In all of these conditions, neither undifferentiated (not shown) nor differentiated MABs showed significant differences in laminin α2 expression (Fig. 3a, b). As expected, as positive control for treatment, MyoG and MyoD expression was increased in MABs treated with AZA and decreased in those treated with TSA, as reported for C2C12 cells (Fig. 3c, d) [42–44].

Finally, we tested whether impaired synthesis of laminin α2 was restricted to mouse MABs. Human MABs were derived from two healthy individuals and expanded in culture. Again, Western blot analysis revealed only minimal (if any) laminin α2 in human MABs (Fig. 3e).

Overall results show that MABs do not synthesize (or they do in minimal amount) laminin-211, suggesting that they may not be sufficient to rescue MDC1A disease.

### Engineered mesoangioblasts can synthesize and secrete miniagrin in muscles of MDC1A mice

**Fig. 4:**
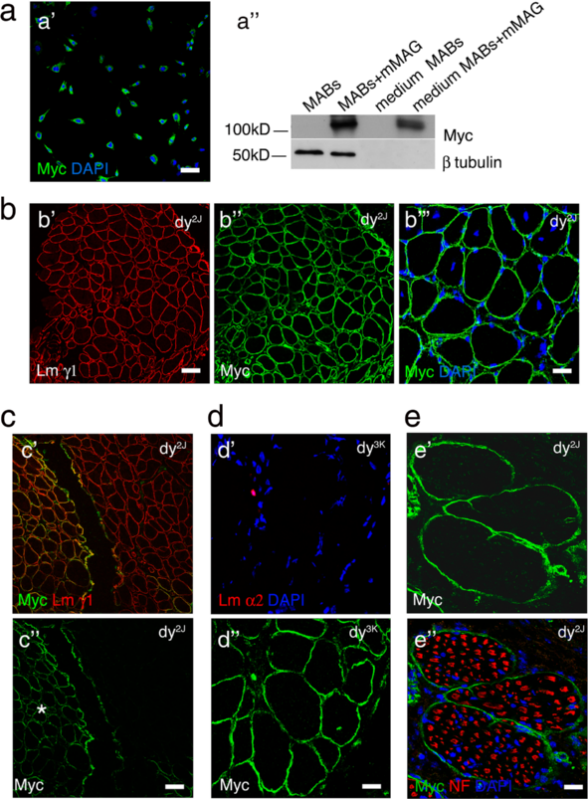
MABs + mMAG can express and synthesize miniagrin and when injected in dy^2J^ colonize the skeletal muscles but not the peripheral nerves. **a** C57 MABs transduced with miniagrin gene carrying myc tag show diffuse positivity when stained with anti-myc antibody (*a* DAPI staining identifies nuclei); scale bar = 30 μm. *b* Western blot analysis with anti-myc antibody, recognizing mMAG, of cell homogenate and medium from cultures of C57 MABs and C57 MABs transduced with miniagrin gene (MABs + mMAG). Anti-myc recognizes a band only in the homogenate and medium of transduced MABs. β-Tubulin is shown as loading control (accordingly, staining is not present in medium). Each lane is loaded with 25 μg of proteins. **b** Cryosection of the tibialis anterior muscle from dy^2J^ mouse treated for 10 days with MABs + mMAG. Double staining with anti-laminin γ1 (*a* depicting myofibers, in *red*) and anti-myc antibody (*b* recognizing mMAG, in *green*) shows diffuse expression of miniagrin around myofibers; scale bar = 50 μm. *c* Same muscle showing myc-positive fibers with centrally located nuclei (identified by DAPI, in *blue*), suggesting regenerating fibers; scale bar = 25 μm. **c** Cryosection of the tibialis anterior muscle from dy^2J^ mouse treated for 10 days with MABs + mMAG. Double staining with anti-laminin γ1 (*a* depicting myofibers, in *red*) and anti-myc antibody (*b* recognizing mMAG, in *green*) shows that myc positivity is not diffuse to the entire muscle but limited to the area of injection (*asterisk*). Scale bar = 50 μm. **d** Cryosection of the tibialis anterior muscle from dy^3K^ mouse 10 days after i.m. injection of MABs carrying a myc tag. Staining with anti-myc antibody recognizes an area where MABs secreted mMAG *a*, whereas staining with anti-laminin α2 antibody does not show positive signal (*b* DAPI staining identifies nuclei). Scale bar = 25 μm. **e** Cryosection of intramuscular nerves of the tibialis anterior muscle from dy^2J^ mouse treated for 10 days with MABs + mMAG. Double staining with neurofilaments (*NF*) recognizing axons *a* and anti-myc antibody *b* recognizing mMAG. Myc staining is restricted to the perineurium and blood vessels, whereas only scarce and scattered positivity is present in the endoneurium. DAPI staining identifies nuclei. Scale bar = 20 μm

As MABs cannot synthesize laminin-211, their potential efficacy in the treatment of MDC1A would be limited. Conversely, the overexpression of *miniagrin* gene was shown to ameliorate the muscular dystrophy in MDC1A mutants. We thus evaluated if the combination of MABs as carrier cells to deliver miniagrin protein into degenerating tissues may ameliorate the phenotype of MDC1A mouse model. An established cell line of mouse MABs (clone C57, [34]) was infected with lentiviral vectors carrying the cDNA of mouse *miniagrin* [20]. By immunohistochemistry, 95 % of infected MABs showed positive staining for miniagrin, recognized by anti-myc antibody (Fig. 4a (a)). We then evaluated whether MABs carrying *miniagrin* (MABs + mMAG) not only synthesize but also secrete miniagrin. MABs + mMAG, and non-infected MABs as control, were grown in petri dishes for 3 days. Medium and cell homogenate was analyzed by Western blotting to investigate the presence of miniagrin. As expected, anti-myc antibody recognized miniagrin in the cell homogenate of MABs + mMAG (Fig. 4a (b)). Similarly, miniagrin was detected in the medium obtained from MABs + mMAG. Lack of β-tubulin confirmed that no (necrotic) cells were present in the medium (Fig. 4a (b)). The homogenate of MABs or medium from MABs loaded as negative control did not show any positive staining for miniagrin (Fig. 4a (b)). These data show that MABs engineered with the *miniagrin* gene can synthesize and secrete the miniagrin protein.

We then evaluated if MABs + mMAG (clone C57) can synthesize and deliver mMAG in the skeletal muscles and peripheral nerves when injected into MDC1A mouse model. To avoid cell rejection, as the MAB C57 clone was allogeneic to mouse mutants, we treated all mice with tacrolimus (FK506), 25 mg/kg/day, starting 3 days before the first cell injection. One million cells (MABs + mMAG, carrying myc tag) were injected intramuscularly into the tibialis anterior and gastrocnemius muscle of three 45-day-old dy^2J^ mice (carrying laminin a2 mutation) and three 18-day-old dy^3K^ mice (completely devoid of laminin α2). Mice were euthanized 10 days after MABs injection, and muscles were stained with anti-myc and anti-laminin antibodies. In both strains (dy^2J^ and dy^3K^), we observed diffuse myc staining around muscle fibers (identified by anti-laminin γ1) in the area of injection (Fig. 4b and data not shown). In many cases, myc-positive fibers showed centrally located nuclei or were NCAM positive, suggesting that they were regenerating fibers (Fig. 4b (c) and not shown). The myc-positive staining was limited to the area of injection, in fact, the rest of the muscle appeared myc-negative (Fig. 4c), and we did not observe myc-positive staining in other muscles within the same leg or in the contralateral leg (data not shown). Interestingly, although we observed myc staining in the injected muscles of dy^3K^ mice, indicating that MABs are present and can synthesize and secrete myc-tagged miniagrin, we did not observe any staining for laminin α2 (Fig. 4d), suggesting that even in vivo MABs cannot synthesize and secrete laminin-211.

Finally, we observed that sciatic nerve branches showed intense myc-positive staining only in the perineurium or around large perineurial blood vessels, whereas only rare myc-positive staining was detected in the endoneurium, suggesting that MABs or delivered miniagrin do not enter the peripheral nerves (Fig. 4e).

Overall, these results show that MABs can deliver miniagrin in vivo, which localizes around muscle fibers in strict correlation with laminins and laminin receptors.

### dy^2J^ Mice treated with MABs + mMAG show better motor performances and ameliorated muscle histology.

We then evaluated whether i.m. injection of MABs and miniagrin delivery may ameliorate muscle histology and motor performances of MDC1A mutants. As this would represent a proof of principle study, and due to lifespan limitation of the dy^3K^ model, we decided to use adult dy^2J^ mice and to inject only few large muscles of the posterior limbs.

**Fig. 5:**
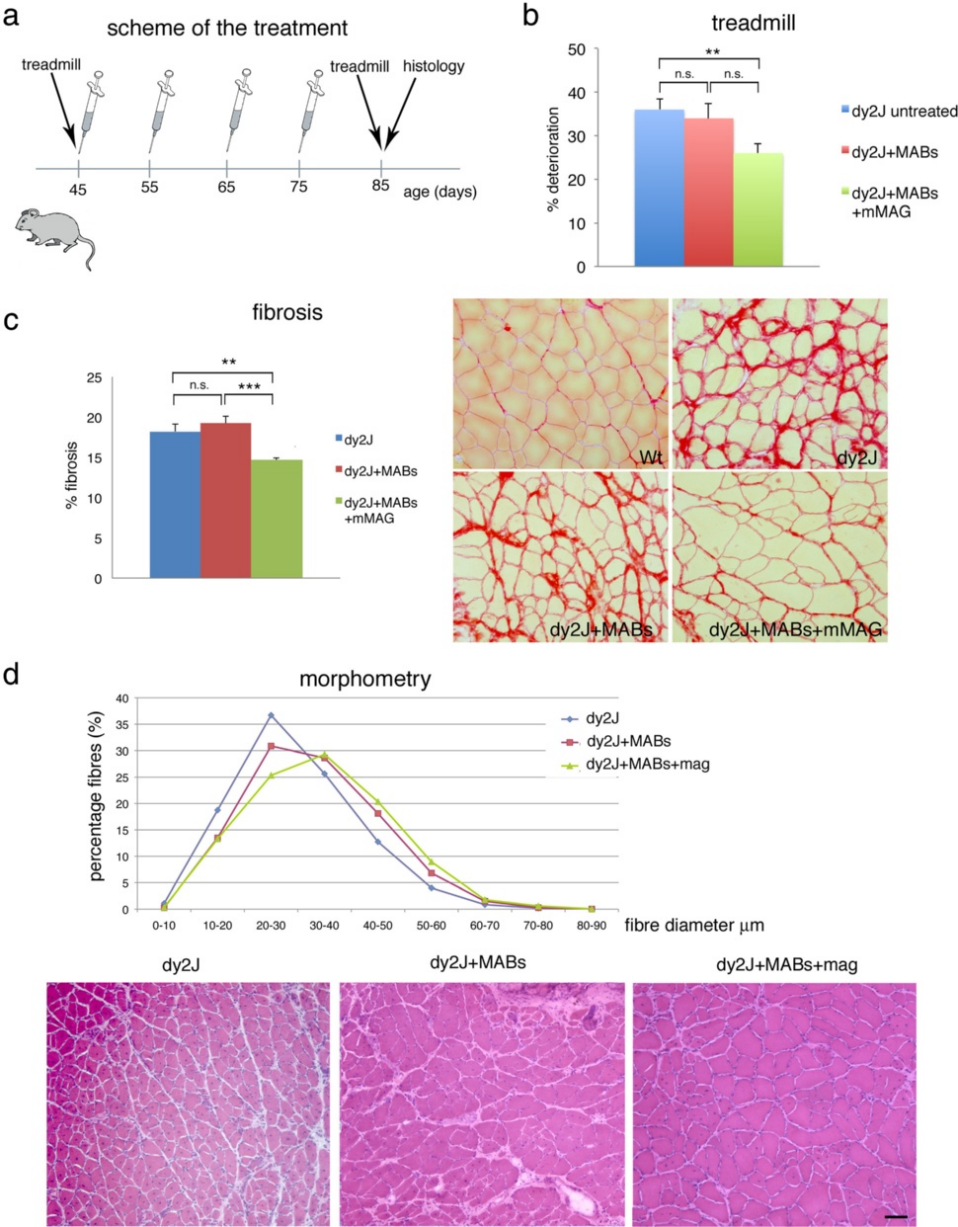
dy^2J^ Mice treated with MABs + mMAG show improved locomotion. **a** Schematic representation of cell treatment, functional, and histological analyses. **b** Treadmill analysis in ten dy^2J^ mice untreated (saline solution, *blue*, group 1), ten treated with MABs + mMAG (*green*, group 2), and ten treated with MABs alone (*red*, group 3) Average times (±SEM) measured on the treadmill before and after treatment are shown on the *left*. On the *right*, the percentage of deterioration between the two measurements is depicted. dy^2J^ Mice treated with MABs + mMAG deteriorate significantly less than untreated dy^2J^ mice (**p < 0.01; *n* = 10; ANOVA). dy^2J^ Mice treated with MABs alone behave similar to controls. **c** Fibrosis revealed by Sirius red staining of the tibialis anterior muscle in dy^2J^ mice untreated (saline solution, *blue*), treated with MABs + mMAG (*green*), and treated with MABs alone (*red*); on the *right*, representative staining of the three groups as compared to normal wild type mice. The area of fibrosis is significantly reduced in dy^2J^ mice treated with MABs + mMAG as compared to dy^2J^ mice treated with saline or MABs alone. **d** Morphometric analysis of the tibialis anterior muscle from dy^2J^ mice untreated (saline, *blue*), treated with MABs + mMAG (*green*), or with MABs alone (*red*). The histogram shows that the fiber size distribution of dy^2J^ mice treated with MABs + mMAG is shifted to the *right* as compared to mice untreated or treated with MABs alone, indicating a higher percentage of fibers with larger diameter. *Below*, the representative H&E staining of muscle histology from the tibialis anterior of the three groups of dy^2J^ mice. Muscle atrophy and endomysial connective tissue is prevalent in untreated muscle and in muscle treated with MABs alone. Reduced muscle fiber size variability and connective tissue is evident in muscle treated with MABs + mMAG. Scale bar = 50 μm

Thirty 45-day-old dy^2J^ mice were divided into three groups: group 1, ten mice were treated with normal saline solution as controls; group 2, ten mice were treated with MABs + mMAG; and group 3, ten mice were treated with MABs (without mMAG). Every mouse received 1.0 × 10^6^ cells by i.m. injection in the tibialis anterior, gastrocnemius, and quadriceps muscle, bilaterally, every 10 days for four times as depicted in Fig. 5a. Motor performances of all mice were evaluated with treadmill test before the first injection (45-day-old) and 10 days after the last injection (85-day-old), and the percentage of deterioration measured (as described in methods). Motor performances of dy^2J^ mice treated with MABs + mMAG deteriorated significantly less than the dy^2J^ mice treated with saline (Fig. 5b; ANOVA 2 tails *p* = 0.01, *n* = 10). Conversely, we did not observe significant differences between dy^2J^ treated with MABs alone (no miniagrin) as compared to those treated with saline (Fig. 5b; *p* = 0.63, *n* = 10).

We then analyzed muscle histology. Fibrosis is a typical feature of dystrophic mice. We evaluated fibrosis by comparing mice in the three groups. Endomysial connective tissue, identified by Sirius Red staining, was measured and expressed as percentage of the evaluated muscle area. We observed a significant decrease of the fibrotic area in dy^2J^ mice treated with MABs + mMAG as compared to dy^2J^-untreated mice (Fig. 5c; ANOVA 2 tails *p* = 0.005, *n* = 5 mice) or mice treated only with MABs (ANOVA 2 tails *p* = 0.0004; *n* = 5 mice).

Mice treated with MABs + mMAG also showed amelioration of muscle histology. In fact, dy^2J^ treated with MABs + mMAG (group 2) showed myofibers with larger diameter as compared to dy^2J^ mice (group 1, treated with saline) and those treated with MABs alone (group 3). We observed in the tibialis anterior muscle a significant shift to the right of the histogram when fibers were plotted against diameters (Fig. 5d), as estimated by comparing differences between data distribution (test for stochastic dominance: *p* < 0.001); similar significant difference was also present between mice treated with MABs + mMAG as compared to those treated with MABs alone (test for stochastic dominance: *p* < 0.001). Accordingly, in the gastrocnemius, the number of fibers of small diameter (10–30 μm) was slightly reduced although not significantly in dy^2J^ mice treated with MABs + mMAG (group 3; 168 ± 50 fibers per 0.1 mm^2^) as compared to dy^2J^ mice treated with saline (group 1; 236 ± 45 fibers per 0.1 mm^2^; *p* = not significant, *n* = 4), whereas the number of fibers of larger diameter (40–60 μm) was significantly increased dy^2J^ mice treated with MABs + mMAG (80 ± 7 fibers per 0.1 mm^2^) as compared to dy^2J^ mice treated with saline (27 ± 9 fibers per 0.1 mm^2^; ANOVA 2 tails, *p* = 0.02; *n* = 4).

When we evaluated the number of fibers with centrally located nuclei, as representative of regenerating fibers to counteract degeneration, we did not observe significant differences between treated and untreated mice: the number of fibers with central nuclei was slightly decreased in dy^2J^ mice treated with MABs + mMAG (31.3 ± 4.2 %) when compared to dy^2J^ mice treated with saline (36.8 ± 3.5 %, ANOVA, *p* = not significant, *n* = 5 per group) or treated with MABs (43.5 ± 3.6 %; ANOVA, *p* = not significant, *n* = 5 per group).

Finally, as apoptosis may contribute to the disease progression of MDC1A [45–47], we evaluated the number of apoptotic muscle fibers by TUNEL labeling assay in treated and untreated dy^2J^ mice. We did not observe significant differences of apoptotic cells in dy^2J^ mice treated with MABs (1.06 ± 0.19 %) versus MABs + mMAG (1.44 ± 0.36 %) or versus untreated dy^2J^ mice (1.26 ± 0.23 %;) as shown in Additional file 1: Figure S1.

### MDC1A mice treated with MABs + mMAG show restored levels of laminin-211 receptors

As laminin-411 and 511 are described upregulated in muscles of Lama2 mice [3, 19, 22, 23], and we further observed that MABs can synthesize laminin chain α4 and α5, we evaluated the expression of these two laminin isoforms in MABs treated and untreated dy^2J^ mice. As expected, dy^2J^ mice showed increased expression of both laminin chain α4 and α5 but we did not observe further differences after treatment with MABs (either MABs alone or MABs + mMAG) as shown in Additional file 2: Figure S2. Interestingly, staining for laminin chain γ1 was changed in dy^2J^ mice as compared to normal controls, as at similar level of detection it was reduced and discontinuous in dy^2J^ mice, with a pattern similar to laminin α5 as shown in Additional file 2: Figure S2C. Treatment with MABs (either alone or MABs + mMAG) did not modify the staining as shown in Additional file 2: Figure S2C. This last data would be consistent with the change of the γ1 chain from the laminin-211 heterotrimer to the 411/511 heterotrimer in dy^2J^ mice.

**Fig. 6:**
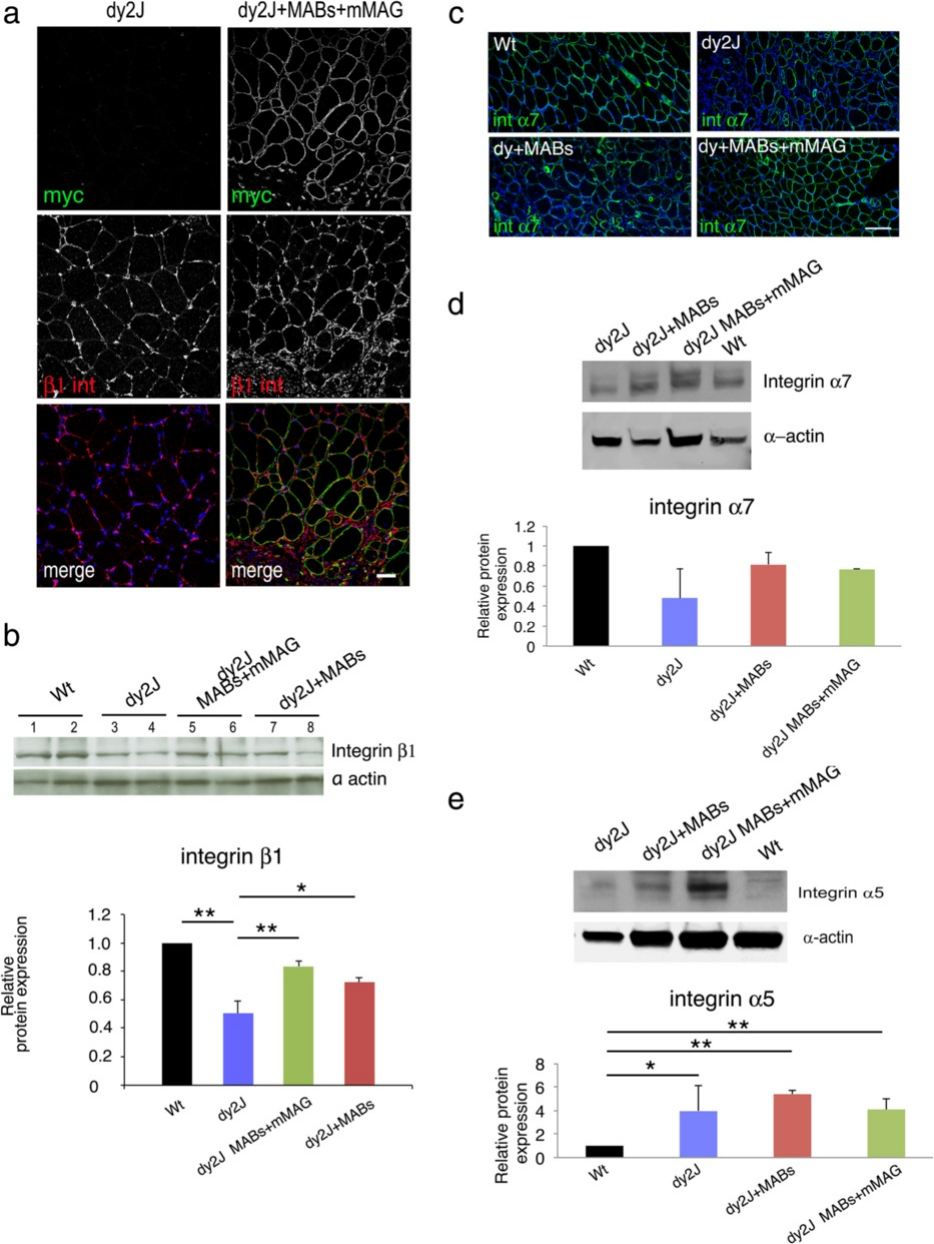
Miniagrin treatment restores expression of β1 integrin receptors. **a** Cryosections of the tibialis anterior muscle from dy^2J^ mice untreated (saline solution) or treated with MABs + mMAG double stained with anti-myc (recognizing mMAG) and anti-β1 integrin antibody. The immunofluorescence shows that myc staining is present only in treated muscle. Staining for β1 integrin is more intense and diffuse in treated muscle. Images are acquired by confocal microscope with the same z-stack and laser intensity. DAPI staining identifies nuclei. **b** Western blot analysis of the tibialis anterior muscle from dy^2J^ mice untreated or treated with MABs + mMAG or MABs alone and stained with anti β1 integrin, and β-actin as loading control. Quantification of Western blot analysis is reported as an average of three independent experiments and represented as the ratios β1 integrin/actin, assigning wild type as 1. A significant decrease of β1 integrin (*n* = 3) is present in muscle of dy^2J^ mice as compare to wild type control; the amount of β1 integrin is significantly increased after treatment with MABs + mMAG. **c** Cryosections of wild type (*Wt*), dy^2J^ untreated (saline), dy^2J^ treated with MABs, and dy^2J^ treated with MABs + mMAG stained with anti α7 integrin antibody and DAPI. Staining for α7 integrin was reduced in dy^2J^ mice as compared to Wt and moderately increased in dy^2J^ mice treated with MABs + mMAG. **d** Western blot analysis confirmed the decrease of α7 integrin in dy^2J^, whereas it was more similar to Wt in dy^2J^ mice treated with MABs or MABs + mMAG; differences were not significant. **e** Western blot analysis of α5 integrin showed a significant increase in all the dy^2J^ mice (treated or not treated) as compared to Wt controls. Scale bar = 50 μm in (**a**) and 100 μm in (**c**). Student *t* test; *p < 0.05; **p < 0.01; *error bars* indicate SEM

It was previously shown that constitutive overexpression of miniagrin in transgenic mice leads to increased levels of laminin receptors [19, 20]. As a further readout of efficacy of our treatment, we thus evaluated if this was also present in dy^2J^ mice treated with MABs + mMAG. First, we investigated the expression of integrin β1, which forms the laminin receptors α6β1 and α7β1. With both immunohistochemistry and Western blotting, we observed that the reduced expression of β1 integrin in muscles of dy^2J^ mice was significantly restored after MAB treatment (either MABs alone or MABs + mMAG; Fig. 6a, b). We observed that also integrin α7 was reduced in dy^2J^ mice and restored after MAB treatment (with similar effect between MABs and MABs + mMAG; Fig. 6c, d). Integrin α6 was not detectable in control muscle (except for blood vessels and intramuscular nerves), whereas faint and dotted staining was observed in some muscle fiber of dy^2J^ mice as shown in Additional file 3: Figure S3. In dy^2J^ mice treated with MABs (either MABs alone or MABs + mMAG), this dotted α6 expression was more evident in the muscle fibers. As integrin α5 is another β1 partner, generating a fibronectin receptor with a relevant role in muscle function, and the balance between laminin and fibronectin receptors is important for muscle integrity [48], we evaluated also α5 expression in dy^2J^-treated mice. The expression of α5 integrin was significantly increased in dy^2J^ mice but not modified after treatment with MABs (either MABs alone or MABs + mMAG; Fig. 6e).

**Fig. 7:**
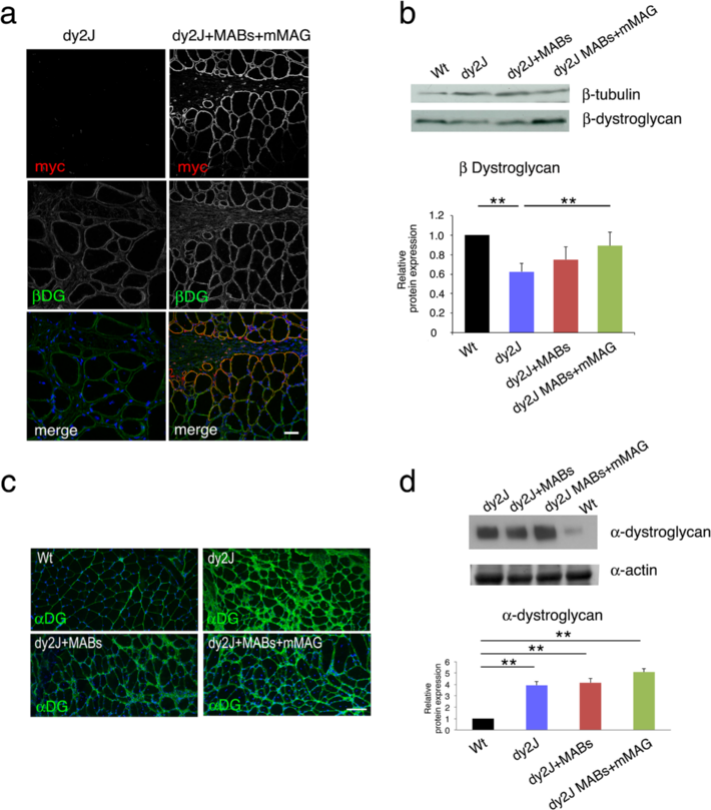
Miniagrin treatment restores expression of dystroglycan receptor in muscles. **a** Cryosections of the tibialis anterior muscle from dy^2J^ mice untreated (saline) or treated with MABs + mMAG double stained with anti-myc (recognizing mMAG) and anti β-dystroglycan integrin antibody. The immunofluorescence shows that myc staining is present only in treated muscle. Staining for β-dystroglycan is more intense and diffuse in treated muscle. Images are acquired by confocal microscope with the same z-stack and laser intensity. DAPI staining identifies nuclei. **b** Western blot analysis of tibialis anterior muscle from dy^2J^ mice untreated, or treated with MABs+mMAG or MABs alone, and and wild type (Wt) controls, stained with anti β-dystroglycan, and β-tubulin as loading control. Quantification of western blot analysis is reported as an average of 3 independent experiments and represented as the ratios β1 integrin/actin, assigning Wt as 1. A significant decrease of β-dystroglycan (n = 3) is present in muscle of dy^2J^ mice as compared to Wt control; the amount of β-dystroglycan is significantly increased after treatment with MABs+mMAG (n = 3). **c** Cryosections of wild type (*Wt*), dy^2J^ untreated (saline), dy^2J^ treated with MABs, and dy^2J^ treated with MABs + mMAG stained with anti α-dystroglycan antibody and DAPI. Staining for α-dystroglycan was increased in dy^2J^ mice as compared to Wt and more similar to Wt control in dy^2J^ mice treated with MABs or MABs + mMAG. Western blot analysis confirmed the significant increase of α-dystroglycan in dy^2J^, as compared to Wt mice; dy^2J^ mice treated with MABs or MABs + mMAG did not show further changes. **d** Western blot analysis of tibialis anterior muscle from dy^2J^ mice untreated, or treated with MABs+mMAG or MABs alone, and Wt controls, stained with anti α-dystroglycan, and β-tubulin as loading control. Quantification of western blot analysis is reported as an average of 3 independent experiments and represented as the ratios β1 integrin/actin, assigning Wt as 1. A significant increase of α-dystroglycan (n = 3) is present in muscle of dy^2J^ mice as compared to Wt control; similar levels are also observed in dy^2J^ mice treated with MABs (either alone or with mMAG; n = 3). Scale bar = 50 μm in **(a)** and 100 μm in **(c)**. Student *t* test; *p < 0.05; **p < 0.01; *error bars* indicate SEM

The other main laminin-211 receptor in the skeletal muscle is the dystroglycan complex, which is formed by α-(extracellular) and β-(transmembrane) subunits [6]. dy^2J^ Mice showed significant reduction of β-dystroglycan, which expression was significantly rescued at normal level after treatment with MABs + mMAG (Fig. 7a, b). Expression of α-dystroglycan was increased in dy^2J^ mice and not changed after MABs treatment (Fig. 7c, d).

### MABs + mMAG treatment ameliorates the clinical phenotype also in SCIDdy^2J^ mice

As a complementary study of the efficacy of MABs + mMAG treatment for MDC1A mouse model, we generated dy^2J^ mice in SCID background (SCIDdy^2J^). The aim of this experiment was to evaluate the efficacy of MAB therapy in the absence of immunoresponse and immune-suppressive therapy. Mice were divided in two groups: group 1 included ten SCIDdy^2J^ mice treated with saline and group 2 included ten SCIDdy^2J^ mice treated with MABs + mMAG. Cell dosage, time points of treatment, and age of treated mice were the same as described above and summarized in Fig. 5a. Motor performances of all mice were evaluated with treadmill just before the first injection (45-day-old), and 10 days after the last injection (85-day-old). As expected, also SCIDdy^2J^ mice treated with MABs + mMAG showed a significant reduction in deterioration of treadmill performances (−29 ± 4 %) as compared to non-treated SCIDdy^2J^ mice (−49 ± 9 %; Student’s *t* test, two tails, *p* = 0.05, *n* = 10), despite extreme variability in genetic background (F3/F4 generation).

### Mesoangioblast treatment did not ameliorate the peripheral neuropathy of MDC1A

Loss of laminin-211 is responsible for dysmyelinating neuropathy in human MDC1A and animal models [17, 18, 49, 50]. Although we did not observe mMAG expression in the endoneurium but it was limited to the perineurium (Fig. 3d), we evaluated whether this was anyway sufficient to modify peripheral neuropathy. Five mice in group 1 (dy^2J^ mice treated with saline solution) and group 2 (dy^2J^ mice treated with MABs + mMAG) also underwent neurophysiological analysis at 45-day-old (before treatment) and 85-day-old (after treatment) to evaluate the effect on the peripheral neuropathy. Neurophysiology of dy^2J^ mice did not show significant differences before and after treatment with MABs + mMAG or saline. Before treatment: group 1 showed cMAP 3.95 ± 0.7 mV and NCV 33 ± 3.5 m/s, whereas group 2 showed cMAP 3.90 ± 0.6 mV and NCV of 32 ± 4 m/s. After treatment: group 1 showed cMAP 3.2 ± 0.7 mV and NCV of 39 ± 2 m/s, whereas group 2 (MABs + mMAG treated) showed cMAP 3.2 ± 0.5 mV and NCV of 38 ± 1.5 m/s (Student’s *t* test, *p* = not significant). Accordingly, the analysis of semithin sections of the sciatic nerves did not show differences between the two groups, as shown in Additional file 4: Figure S4.

Overall, these data show that mMAG delivered by MABs injected into the skeletal muscles does not enter significantly in the peripheral nerves, and this therapy does not interfere with the progression of the peripheral neuropathy in MDC1A mutants.

## Discussion

We propose to associate cell (MABs) and gene (mMAG) therapy as a strategy to treat MDC1A. Results of our proof of principle study are consistent with an amelioration of morphological, molecular, and motor aspects of MDC1A-treated mice. No effect was observed on the associated peripheral neuropathy.

Although previous studies showed that MAB cell therapy is sufficient to treat recessive muscular dystrophies in preclinical models [32–36, 41], this was not considered a successful strategy in MDC1A as we observed that MABs synthesize and secrete only little (negligible) amount of laminin α2, which is the missing protein in MDC1A. Lack of laminin α2 synthesis was not restricted to mouse species, as we observed similar result with human MABs. It was even independent of epigenetic events, as neither inhibition of histone deacetylation nor of DNA methylation induced murine MABs to synthesize more laminin α2. Although this seems contradictory with the fact that MABs differentiate into myocytes, which normally produce laminin α2, and that once differentiation is triggered it will automatically lead to the full expression of the gene repertoire, this is not necessarily true. It is well known that myoblast differentiation in vitro will not give rise to the expression of adult isoforms of sarcomeric proteins [51]. Nonetheless, previous in vivo studies showed that bone marrow-derived stem cells differentiated into myocytes could not synthesize δ-sarcoglycan [52].

Thus, we decided to use MABs as carrier cells to deliver in the skeletal muscle an exogenous protein, miniagrin, which is able to link laminin-211 receptors with other laminin isoforms expressed (or even overexpressed) in the extracellular matrix of MDC1A dystrophic mice [53]. Interestingly, we showed that MABs + mMAG could profusely synthesize these other laminin isoforms, thus increasing the potential efficacy of this strategy. As expected, we observed diffuse staining for mMAG (recognized by anti-myc tag antibody) in the injected area of the skeletal muscles, which associated with signs of regeneration (centrally located nuclei and NCAM staining) when detected few days after injection, and amelioration of morphological (reduction of endomysial connective tissue and increased proportion of fibers with large diameter, suggesting muscle reconstitution and reduced fiber degeneration) and molecular (increased levels of laminin-211 receptors) features when investigated at the end of the preclinical trial. Interestingly, and consistently with previous reports [19–21], it seems that both dystroglycan and α7β1 integrin are the two laminin receptors rescued by MABs + mMAG treatment, as instead α6(β1), which forms the other main integrin-laminin receptor in the muscle [54], was not significantly upregulated. Moreover, α- and β-dystroglycan were differently regulated in dy^2J^ mice, but this is not new as observed also in other Lama2 strains [19, 21], possibly due to different regulatory mechanisms for the two subunits when the ligand is absent (as in dy^3K^ or dy^W^) or not functioning (as in dy^2J^ mice).

We did not investigate the extent of MABs (and MABs + mMAG) engraftment in the injected skeletal muscles and that of MABs integration in regenerating fibers, as (1) it was diffusely proven and shown in previous published studies [32–36, 41, 55]: MABs can fuse with regenerating myotubes and restore missing proteins in loss-of-function myopathies; (2) this was not the scope of our study, as our goal was to demonstrate that injected MABs can secrete MAG in the skeletal muscles (recognized by the myc tag). Moreover, we had no useful marker to differentiate donor from endogenous nuclei in myotubes of injected MDC1A muscles. However, we may speculate that a consistent number of MABs + mMAG have been integrated into the host MDC1A muscles. This would be in line with previous published studies (as reported above), would sustain diffuse and prolonged miniagrin staining around muscle fibers of injected muscles (and not elsewhere) and would justify the enhanced expression of laminin-211 receptors (dystroglycan and integrin α7β1) in MAB + mMAG injected muscles.

MABs + mMAG-treated dy^2J^ mice showed a significant reduction in the deterioration of motor performances with the treadmill analysis as compared to those treated with saline solution, whereas dy^2J^ mice treated with MABs alone did not behave better than those treated with saline. Although MABs + mMAG-treated dy^2J^ mice did not rescue motor performances towards normal values, this was expected as we injected only few muscles of the posterior limbs of dystrophic mice, and this therapy did not interfere with the ongoing peripheral neuropathy. In fact, this would simply constitute a proof-of-concept study on the feasibility of MABs + mMAG therapy for MDC1A. Nonetheless, systemic administration of MABs + mMAG through the arterial vascular system would constitute the gold standard for future translational applications in MDC1A, as already shown in other models [32, 36, 55], and recently in humans (EudraCT no. 2011–000176–33; Giulio Cossu, manuscript in preparation). In fact, MABs can cross the vessel wall and distribute into the dystrophic muscles when delivered intra-arterially. Unfortunately, repetitive intra-arterial injection of MABs in dy^2J^ mice was not feasible for unexpected arterial fragility. Moreover, efficacy of MABs + mMAG treatment in our study was limited to dy^2J^ mice, as more severe model as dy^3K^ die very early due to respiratory failure and we had no chance to deliver engineered MABs directly into the respiratory muscles.

Although MABs are not known to generate neural cell types, as carrier cells, they may enter several tissues and organs and may secrete mMAG in the peripheral nerves, the other target organ of MDC1A. A progressive severe dysmyelinating neuropathy is in fact associated to MDC1A in humans and mouse models [17, 18, 49, 50], and it is already known that genetic repletion of laminin chain α2 in the skeletal muscles of mouse mutants does not completely revert the clinical phenotype due to the progression of the peripheral neuropathy [56]. Hence, we evaluated whether MABs may engraft the peripheral nerves, secrete miniagrin, and possibly ameliorate the peripheral neuropathy associated to MDC1A. Unfortunately, only few MABs entered the endoneurium, while most were blocked in the perineurium of peripheral nerves. This was not sufficient to produce enough miniagrin to change the neuropathy outcome, as demonstrated by pathological and neurophysiological evaluations. Part of this inefficient engraftment could also be due to the presence of blood-nerve barrier. MABs administration in mouse models with disrupted blood-nerve barrier, as following nerve crush injury, showed much higher MABs engraftment in the sciatic nerve endoneurium (Stefano C. Previtali, unpublished data). As other stem cell types have been shown to be better carrier cells for peripheral nerve, i.e., hematopoietic stem cells [29], future studies using combinatory cell therapies such as hematopoietic stem cells to carry miniagrin in the peripheral nerves and MABs + mMAG for the skeletal muscle may constitute an ideal approach for multiorgan disorders such as MDC1A.

## Conclusions

The combination of cell plus gene therapy by MABs engineered to produce and secrete MAG into the skeletal muscles of MDC1A mutants showed significant efficacy to ameliorate muscular dystrophy and motor performances of dystrophic mice. This approach may speed translational studies on the efficacy of miniagrin in human MDC1A, as both MABs delivery and lentiviral transduced cells have been already used in human clinical trials.

## Additional files

Additional file 1: Figure S1. Quantification of TUNEL assay in sciatic nerves section of dy^2J^ mice untreated, treated with MABs, or with MABs + mMAG. (A) TUNEL staining (green signal) in representative sections of the three treatment conditions did not show significant differences (ANOVA, *n* = 3) as reported in quantitative analysis (B). (TIFF 1.77 mb) Additional file 2: Figure S2. Expression of laminin-411 and -511 in treated and not-treated dy^2J^ mice. (A) Western blot analysis of the tibialis anterior muscle from wild type (Wt), dy^2J^ mice untreated (saline) or dy^2J^ mice treated with MABs + mMAG or MABs alone stained with anti α4 laminin antibody, and α-actin as loading control. Quantification of Western blot analysis is reported as an average of two independent experiments and represented as the ratios α4 laminin/actin, assigning wild type as 1. Laminin chain α4 is increased in muscle of dy^2J^ mice, either untreated or treated with MABs or MABs + mMAG, as compare to Wt control. (B) Cryosections of the tibialis anterior muscle from Wt, dy^2J^ mice untreated (saline solution) or treated xwith MABs + mMAG stained with anti α5 laminin antibody. The immunofluorescence shows that staining for α5 laminin is increased in dy^2J^ and dy^2J^ treated with MABs + mMAG muscle as compared to Wt. Images are acquired by confocal microscope, one single section with the same laser intensity. DAPI staining identifies nuclei. (C) Cryosections of the tibialis anterior muscle from Wt, dy^2J^ mice untreated (saline solution) or treated with MABs + mMAG stained with anti γ1 laminin antibody. The immunofluorescence shows that staining for γ1 laminin is reduced in muscle of dy^2J^ and dy^2J^ mice treated with MABs + mMAG as compared to Wt. Images are acquired by confocal microscope, one single section with the same laser intensity. DAPI staining identifies nuclei. Scale bar = 100 μm. (TIFF 2.41 mb) Additional file 3: Figure S3. Expression of integrin α6 in treated and not-treated dy^2J^ mice. Cryosections of the tibialis anterior muscle from Wt, dy^2J^ mice untreated (saline solution) or treated with MABs + mMAG stained with anti α6 integrin antibody. The immunofluorescence shows positive staining limited to the nerves and blood vessels in Wt skeletal muscle. In dy^2J^ mice, faint and dotted staining for α6 integrin in the skeletal muscle was observed, as it was after mesoangioblast treatment (either MABs alone or MABs + mMAG). DAPI staining identifies nuclei. Scale bar = 100 μm. (TIFF 1.05 mb) Additional file 4: Figure S4. Sciatic nerve of treated and not-treated dy^2J^ mice. (A) Semithin section of the sciatic nerve of not-treated (saline solution) dy^2J^ mouse 85-day-old showing bundles of unsorted axons and hypomyelinated fibers. (B) Semithin section of the sciatic nerve of treated (MABs + mMAG) dy^2J^ mouse 85-day-old showing bundles of unsorted axons and hypomyelinated fibers. (TIFF 1.59 mb)

## Abbreviations

cMAP: compound motor action potential
DMEM: Dulbecco’s modified Eagle’s medium
i.m.: intramuscular
MAB: mesoangioblast
MAG: miniagrin
MDC1A: merosin-deficient congenital muscular dystrophy type 1A
MyHC: myosin heavy chain
NCV: nerve conduction velocity

## References

[1] Voit T, Tome F, Engel A, Franzini-Armstrong C (2004). The congenital muscular dystrophies. Myology.

[2] Jimenez-Mallebrera C, Brown SC, Sewry CA, Muntoni F (2005). Congenital muscular dystrophy: molecular and cellular aspects. Cell Mol Life Sci.

[3] Patton B, Miner J, Chiu A, Sanes J (1997). Distribution and function of laminins in the neuromuscular system of developing, adult, and mutant mice. J Cell Biol.

[4] Previtali SC, Nodari A, Taveggia C, Pardini C, Dina G, Villa A, Wrabetz L, Quattrini A, Feltri ML (2003). Expression of laminin receptors in schwann cell differentiation: evidence for distinct roles. J Neurosci.

[5] Durbeej M (2010). Laminins. Cell Tissue Res.

[6] Colognato H, Winkelmann D, Yurchenco P (1999). Laminin polymerization induces a receptor-cytoskeleton network. J Cell Biol.

[7] Mayer U, Saher G, Fassler R, Bornemann A, Echtermeyer F, von der Mark H, Miosge N, Poschl E, von der Mark K (1997). Absence of integrin alpha 7 causes a novel form of muscular dystrophy. Nat Genet.

[8] Feltri M, Graus-Porta D, Previtali S, Nodari A, Migliavacca B, Cassetti A, Littlewood-Evans A, Reichardt L, Messing A, Quattrini A (2002). Conditional disruption of beta1 integrin in Schwann cells impedes interactions with axons. J Cell Biol.

[9] Saito F, Moore SA, Barresi R, Henry MD, Messing A, Ross-Barta SE, Cohn RD, Williamson RA, Sluka KA, Sherman DL (2003). Unique role of dystroglycan in peripheral nerve myelination, nodal structure, and sodium channel stabilization. Neuron.

[10] Pellegatta M, De Arcangelis A, D’Urso A, Nodari A, Zambroni D, Ghidinelli M, Matafora V, Williamson C, Georges-Labouesse E, Kreidberg J (2013). alpha6beta1 and alpha7beta1 integrins are required in Schwann cells to sort axons. J Neurosci.

[11] Previtali SC, Dina G, Nodari A, Fasolini M, Wrabetz L, Mayer U, Feltri ML, Quattrini A (2003). Schwann cells synthesize alpha7beta1 integrin which is dispensable for peripheral nerve development and myelination. Mol Cell Neurosci.

[12] Schwander M, Leu M, Stumm M, Dorchies OM, Ruegg UT, Schittny J, Muller U (2003). Beta1 integrins regulate myoblast fusion and sarcomere assembly. Dev Cell.

[13] Cohn RD, Henry MD, Michele DE, Barresi R, Saito F, Moore SA, Flanagan JD, Skwarchuk MW, Robbins ME, Mendell JR (2002). Disruption of DAG1 in differentiated skeletal muscle reveals a role for dystroglycan in muscle regeneration. Cell.

[14] Meier H, Southard JL (1970). Muscular dystrophy in the mouse caused by an allele at the dy-locus. Life Sci.

[15] Xu H, Wu X-R, Wewer U, Engvall E (1994). Murine muscular dystrophy caused by a mutation in the laminin alpha2 (Lama2) gene. Nature Genet.

[16] Sunada Y, Bernier S, Utani A, Yamada Y, Campbell K (1995). Identification of a novel mutant transcript of laminin alpha2 chain gene responsible for muscular dystrophy and dysmyelination in dy2J mice. Hum Mol Genet.

[17] Miyagoe Y, Hanaoka K, Nonaka I, Hayasaka M, Nabeshima Y, Arahata K, Nabeshima Y, Takeda S (1997). Laminin alpha2 chain-null mutant mice by targeted disruption of the lama2 gene: a new model of merosin (laminin 2)-deficient congenital muscular dystrophy. FEBS.

[18] Guo LT, Zhang XU, Kuang W, Xu H, Liu LA, Vilquin JT, Miyagoe-Suzuki Y, Takeda S, Ruegg MA, Wewer UM (2003). Laminin alpha2 deficiency and muscular dystrophy; genotype-phenotype correlation in mutant mice. Neuromuscul Disord.

[19] Moll J, Barzaghi P, Lin S, Bezakova G, Lochmuller H, Engvall E, Muller U, Ruegg MA (2001). An agrin minigene rescues dystrophic symptoms in a mouse model for congenital muscular dystrophy. Nature.

[20] Meinen S, Barzaghi P, Lin S, Lochmuller H, Ruegg MA (2007). Linker molecules between laminins and dystroglycan ameliorate laminin-alpha2-deficient muscular dystrophy at all disease stages. J Cell Biol.

[21] Bentzinger CF, Barzaghi P, Lin S, Ruegg MA (2005). Overexpression of mini-agrin in skeletal muscle increases muscle integrity and regenerative capacity in laminin-alpha2-deficient mice. FASEB J.

[22] Talts JF, Sasaki T, Miosge N, Gohring W, Mann K, Mayne R, Timpl R (2000). Structural and functional analysis of the recombinant G domain of the laminin alpha4 chain and its proteolytic processing in tissues. J Biol Chem.

[23] Ringelmann B, Roder C, Hallmann R, Maley M, Davies M, Grounds M, Sorokin L (1999). Expression of laminin alpha1, alpha2, alpha4, and alpha5 chains, fibronectin, and tenascin-C in skeletal muscle of dystrophic 129ReJ dy/dy mice. Exp Cell Res.

[24] Qiao C, Li J, Zhu T, Draviam R, Watkins S, Ye X, Chen C, Xiao X (2005). Amelioration of laminin-alpha2-deficient congenital muscular dystrophy by somatic gene transfer of miniagrin. Proc Natl Acad Sci U S A.

[25] Ginn SL, Alexander IE, Edelstein ML, Abedi MR, Wixon J (2013). Gene therapy clinical trials worldwide to 2012—an update. J Gene Med.

[26] O’Reilly M, Federoff HJ, Fong Y, Kohn DB, Patterson AP, Ahmed N, Asokan A, Boye SE, Crystal RG, De Oliveira S (2014). Gene therapy: charting a future course—summary of a National Institutes of Health Workshop, April 12, 2013. Hum Gene Ther.

[27] Meng J, Muntoni F, Morgan JE (2011). Stem cells to treat muscular dystrophies—where are we?. Neuromuscul Disord.

[28] Partridge TA (2011). Impending therapies for Duchenne muscular dystrophy. Curr Opin Neurol.

[29] Biffi A, De Palma M, Quattrini A, Del Carro U, Amadio S, Visigalli I, Sessa M, Fasano S, Brambilla R, Marchesini S (2004). Correction of metachromatic leukodystrophy in the mouse model by transplantation of genetically modified hematopoietic stem cells. J Clin Invest.

[30] Biffi A, Montini E, Lorioli L, Cesani M, Fumagalli F, Plati T, Baldoli C, Martino S, Calabria A, Canale S (2013). Lentiviral hematopoietic stem cell gene therapy benefits metachromatic leukodystrophy. Science.

[31] Minasi MG, Riminucci M, De Angelis L, Borello U, Berarducci B, Innocenzi A, Caprioli A, Sirabella D, Baiocchi M, De Maria R (2002). The meso-angioblast: a multipotent, self-renewing cell that originates from the dorsal aorta and differentiates into most mesodermal tissues. Development.

[32] Sampaolesi M, Blot S, D’Antona G, Granger N, Tonlorenzi R, Innocenzi A, Mognol P, Thibaud JL, Galvez BG, Barthelemy I (2006). Mesoangioblast stem cells ameliorate muscle function in dystrophic dogs. Nature.

[33] Sampaolesi M, Torrente Y, Innocenzi A, Tonlorenzi R, D’Antona G, Pellegrino MA, Barresi R, Bresolin N, De Angelis MG, Campbell KP (2003). Cell therapy of alpha-sarcoglycan null dystrophic mice through intra-arterial delivery of mesoangioblasts. Science.

[34] Diaz-Manera J, Touvier T, Dellavalle A, Tonlorenzi R, Tedesco FS, Messina G, Meregalli M, Navarro C, Perani L, Bonfanti C (2010). Partial dysferlin reconstitution by adult murine mesoangioblasts is sufficient for full functional recovery in a murine model of dysferlinopathy. Cell Death Dis.

[35] Berry SE, Liu J, Chaney EJ, Kaufman SJ (2007). Multipotential mesoangioblast stem cell therapy in the mdx/utrn−/− mouse model for Duchenne muscular dystrophy. Regen Med.

[36] Tedesco FS, Gerli MF, Perani L, Benedetti S, Ungaro F, Cassano M, Antonini S, Tagliafico E, Artusi V, Longa E (2012). Transplantation of genetically corrected human iPSC-derived progenitors in mice with limb-girdle muscular dystrophy. Sci Transl Med.

[37] Tonlorenzi R, Dellavalle A, Schnapp E, Cossu G, Sampaolesi M (2007). Isolation and characterization of mesoangioblasts from mouse, dog, and human tissues. Curr Protoc Stem Cell Biol.

[38] Triolo D, Dina G, Lorenzetti I, Malaguti M, Morana P, Del Carro U, Comi G, Messing A, Quattrini A, Previtali SC (2006). Loss of glial fibrillary acidic protein (GFAP) impairs Schwann cell proliferation and delays nerve regeneration after damage. J Cell Sci.

[39] Porrello E, Rivellini C, Dina G, Triolo D, Del Carro U, Ungaro D, Panattoni M, Feltri ML, Wrabetz L, Pardi R (2014). Jab1 regulates Schwann cell proliferation and axonal sorting through p27. J Exp Med.

[40] Benjamini Y, Hochberg Y (1995). Controlling the false discovery rate: a practical and powerful approach to multiple testing. J R Stat Soc Ser B.

[41] Fuoco C, Salvatori ML, Biondo A, Shapira-Schweitzer K, Santoleri S, Antonini S, Bernardini S, Tedesco FS, Cannata S, Seliktar D (2012). Injectable polyethylene glycol-fibrinogen hydrogel adjuvant improves survival and differentiation of transplanted mesoangioblasts in acute and chronic skeletal-muscle degeneration. Skelet Muscle.

[42] Montesano A, Luzi L, Senesi P, Terruzzi I (2013). Modulation of cell cycle progression by 5-azacytidine is associated with early myogenesis induction in murine myoblasts. Int J Biol Sci.

[43] Hupkes M, Jonsson MK, Scheenen WJ, van Rotterdam W, Sotoca AM, van Someren EP, van der Heyden MA, van Veen TA, van Ravestein-van Os RI, Bauerschmidt S (2011). Epigenetics: DNA demethylation promotes skeletal myotube maturation. Faseb J.

[44] Hagiwara H, Saito F, Masaki T, Ikeda M, Nakamura-Ohkuma A, Shimizu T, Matsumura K (2011). Histone deacetylase inhibitor trichostatin A enhances myogenesis by coordinating muscle regulatory factors and myogenic repressors. Biochem Biophys Res Commun.

[45] Carmignac V, Quere R, Durbeej M (2011). Proteasome inhibition improves the muscle of laminin alpha2 chain-deficient mice. Hum Mol Genet.

[46] Girgenrath M, Dominov JA, Kostek CA, Miller JB (2004). Inhibition of apoptosis improves outcome in a model of congenital muscular dystrophy. J Clin Invest.

[47] Yu Q, Sali A, Van der Meulen J, Creeden BK, Gordish-Dressman H, Rutkowski A, Rayavarapu S, Uaesoontrachoon K, Huynh T, Nagaraju K (2013). Omigapil treatment decreases fibrosis and improves respiratory rate in dy(2J) mouse model of congenital muscular dystrophy. PLoS ONE.

[48] Mayer U (2003). Integrins: redundant or important players in skeletal muscle?. J Biol Chem.

[49] Di Muzio A, De Angelis MV, Di Fulvio P, Ratti A, Pizzuti A, Stuppia L, Gambi D, Uncini A (2003). Dysmyelinating sensory-motor neuropathy in merosin-deficient congenital muscular dystrophy. Muscle Nerve.

[50] Shorer Z, Philpot J, Muntoni F, Sewry C, Dubowitz V (1995). Demyelinating peripheral neuropathy in merosin-deficient congenital muscular dystrophy. J Child Neurol.

[51] Weiss A, Leinwand LA (1996). The mammalian myosin heavy chain gene family. Annu Rev Cell Dev Biol.

[52] Lapidos KA, Chen YE, Earley JU, Heydemann A, Huber JM, Chien M, Ma A, McNally EM (2004). Transplanted hematopoietic stem cells demonstrate impaired sarcoglycan expression after engraftment into cardiac and skeletal muscle. J Clin Invest.

[53] Meinen S, Ruegg MA (2006). Congenital muscular dystrophy: mini-agrin delivers in mice. Gene Ther.

[54] Meyer U (2003). Integrins: redundant or important players in skeletal muscle. J Biol Chem.

[55] Tedesco FS, Hoshiya H, D’Antona G, Gerli MF, Messina G, Antonini S, Tonlorenzi R, Benedetti S, Berghella L, Torrente Y (2011). Stem cell-mediated transfer of a human artificial chromosome ameliorates muscular dystrophy. Sci Transl Med.

[56] Kuang W, Xu H, Vachon P, Liu L, Loechel F, Wewer U, Engvall E (1998). Merosin-deficient congenital muscular dystrophy. J Clin Invest.

