# Supplementary figures and images for: Mesoangioblast delivery of miniagrin ameliorates murine model of merosin-deficient congenital muscular dystrophy type 1A
Source: Skelet Muscle. 2015 Sep 3;5:30. doi: 10.1186/s13395-015-0055-5 (PMC4560053; doi:10.1186/s13395-015-0055-5)

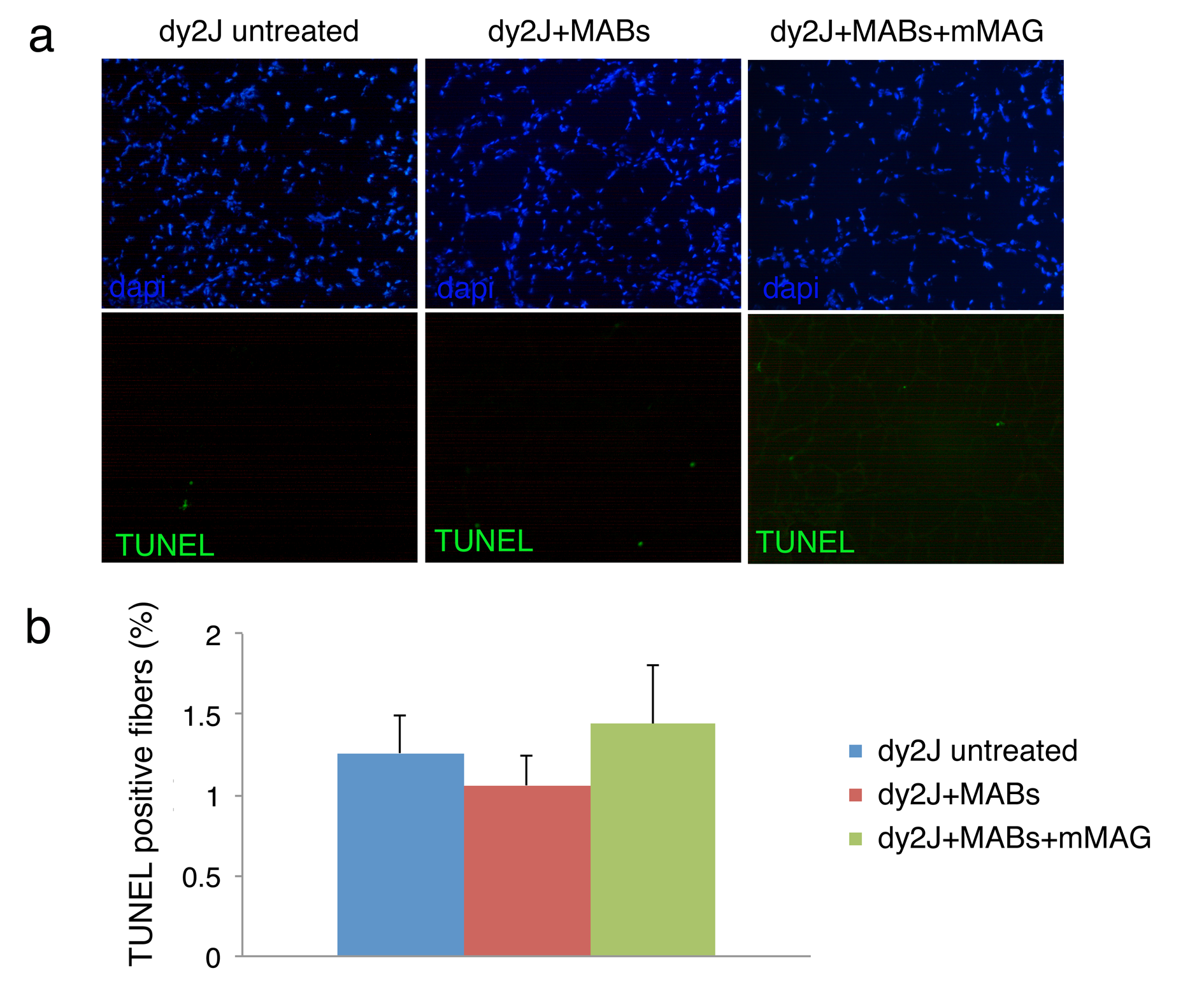

Supplement: Additional file 1: Figure S1. — Quantification of TUNEL assay in sciatic nerves section of dy2J mice untreated, treated with MABs, or with MABs + mMAG. (A) TUNEL staining (green signal) in representative sections of the three treatment conditions did not show significant differences (ANOVA, n = 3) as reported in quantitative analysis (B). (TIFF 1.77 mb) [file 13395_2015_55_MOESM1_ESM.tif]

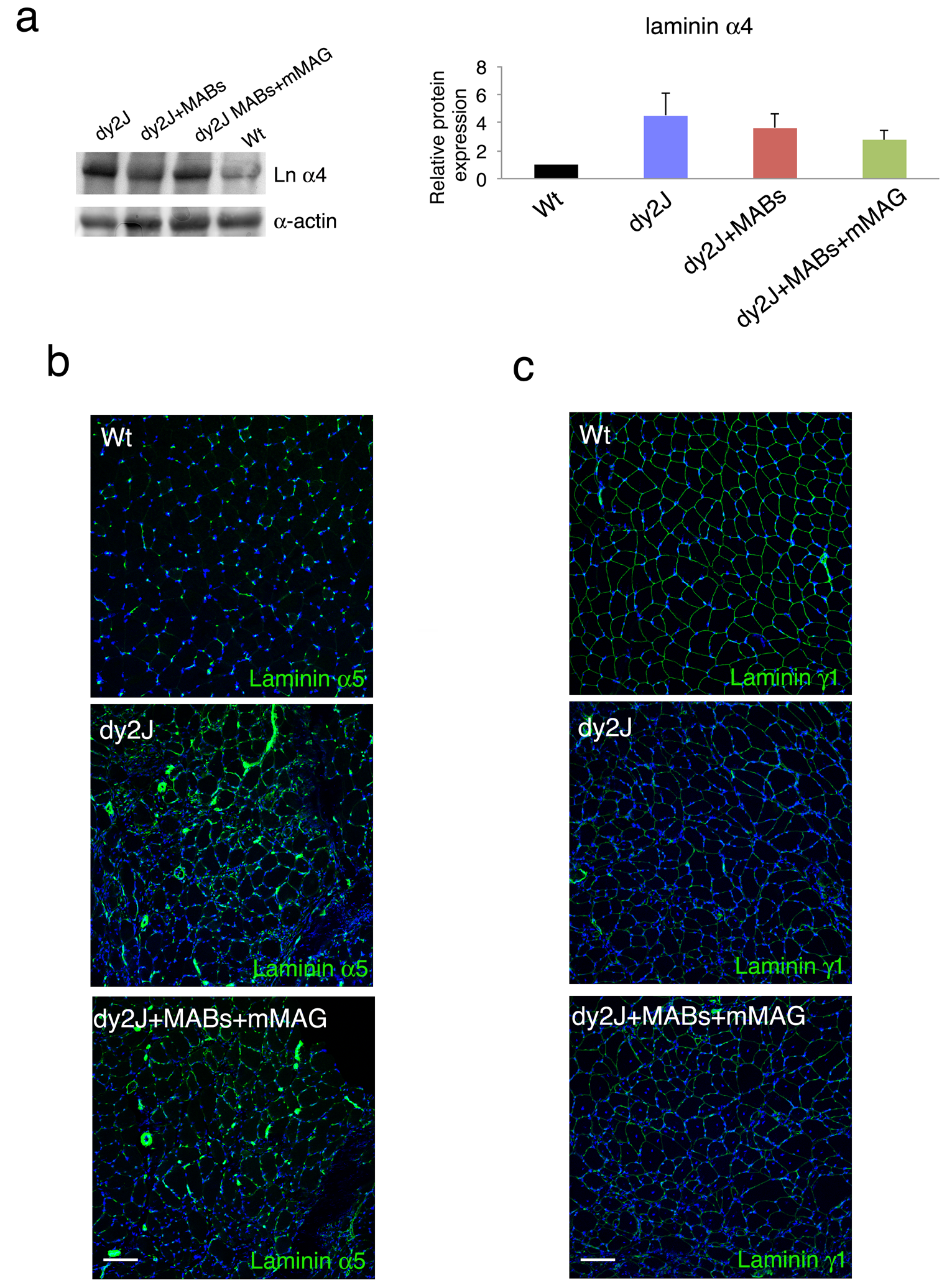

Supplement: Additional file 2: Figure S2. — Expression of laminin-411 and -511 in treated and not-treated dy2J mice. (A) Western blot analysis of the tibialis anterior muscle from wild type (Wt), dy2J mice untreated (saline) or dy2J mice treated with MABs + mMAG or MABs alone stained with anti α4 laminin antibody, and α-actin as loading control. Quantification of Western blot analysis is reported as an average of two independent experiments and represented as the ratios α4 laminin/actin, assigning wild type as 1. Laminin chain α4 is increased in muscle of dy2J mice, either untreated or treated with MABs or MABs + mMAG, as compare to Wt control. (B) Cryosections of the tibialis anterior muscle from Wt, dy2J mice untreated (saline solution) or treated xwith MABs + mMAG stained with anti α5 laminin antibody. The immunofluorescence shows that staining for α5 laminin is increased in dy2J and dy2J treated with MABs + mMAG muscle as compared to Wt. Images are acquired by confocal microscope, one single section with the same laser intensity. DAPI staining identifies nuclei. (C) Cryosections of the tibialis anterior muscle from Wt, dy2J mice untreated (saline solution) or treated with MABs + mMAG stained with anti γ1 laminin antibody. The immunofluorescence shows that staining for γ1 laminin is reduced in muscle of dy2J and dy2J mice treated with MABs + mMAG as compared to Wt. Images are acquired by confocal microscope, one single section with the same laser intensity. DAPI staining identifies nuclei. Scale bar = 100 μm. (TIFF 2.41 mb) [file 13395_2015_55_MOESM2_ESM.tif]

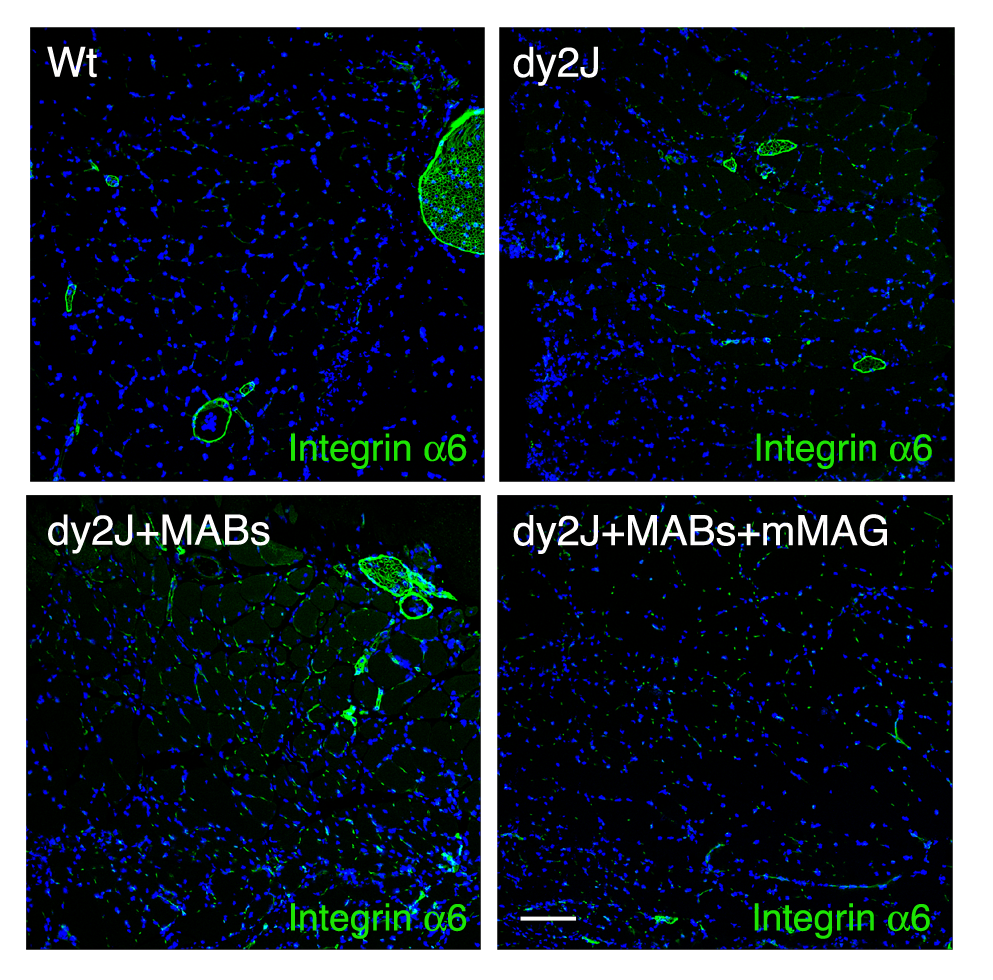

Supplement: Additional file 3: Figure S3. — Expression of integrin α6 in treated and not-treated dy2J mice. Cryosections of the tibialis anterior muscle from Wt, dy2J mice untreated (saline solution) or treated with MABs + mMAG stained with anti α6 integrin antibody. The immunofluorescence shows positive staining limited to the nerves and blood vessels in Wt skeletal muscle. In dy2J mice, faint and dotted staining for α6 integrin in the skeletal muscle was observed, as it was after mesoangioblast treatment (either MABs alone or MABs + mMAG). DAPI staining identifies nuclei. Scale bar = 100 μm. (TIFF 1.05 mb) [file 13395_2015_55_MOESM3_ESM.tif]

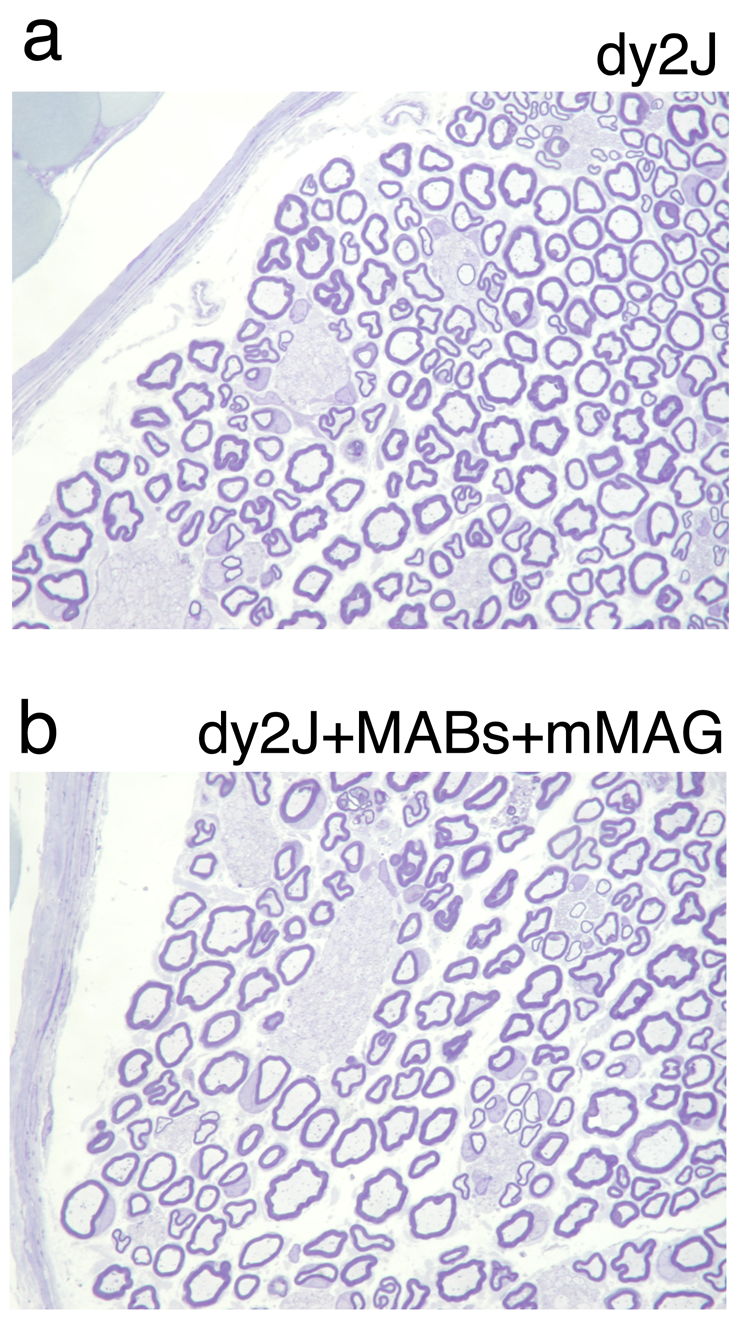

Supplement: Additional file 4: Figure S4. — Sciatic nerve of treated and not-treated dy2J mice. (A) Semithin section of the sciatic nerve of not-treated (saline solution) dy2J mouse 85-day-old showing bundles of unsorted axons and hypomyelinated fibers. (B) Semithin section of the sciatic nerve of treated (MABs + mMAG) dy2J mouse 85-day-old showing bundles of unsorted axons and hypomyelinated fibers. (TIFF 1.59 mb) [file 13395_2015_55_MOESM4_ESM.tif]
